# An Update on Breakthrough Invasive Mold Infections

**DOI:** 10.1007/s11046-024-00864-z

**Published:** 2024-06-13

**Authors:** Vera Portillo, Dionysios Neofytos

**Affiliations:** 1 Division of Infectious Diseases, University Hospital of Geneva, Rue Gabrielle-Perret-Gentil 4, 1211 Geneva, Switzerland; 2 Internal Medecine, Ensemble Hospitalier de la Côte, Hôpital de Moges, Chemin de la Crêt 2, Morges, Vaud, Switzerland

**Keywords:** Breakthrough fungal infections, Posaconazole, Breakthrough mold infections

## Abstract

The incidence of breakthrough mold infections (bIMI) has been increasing, due to routine administration of broad-spectrum antifungal prophylaxis and an increasing pool of high-risk patient populations, with fungi more challenging to treat, resulting in a sustained high mortality, despite progress in diagnostic and therapeutic options. Pharmacokinetics of antifungal drugs, fungal, and host, including genetic, factors play a role in the emergence of bIMI. Suggested therapeutic approaches have included change of antifungal class treatment, with amphotericin-B products predominating as first-line empirical treatment and switching from one broad-spectrum azole to another remaining the most frequently used treatment modalities. Future perspectives include determining individual susceptibility to IMI to tailor prophylaxis and treatment strategies, improved diagnostic tests, and the introduction of new antifungal agents that may reduce morbidity and mortality caused by bIMI.

## Introduction

Invasive mold infections (IMIs) are a major cause of mortality among immunosuppressed hosts [1–5]. Despite progress in diagnostics, treatment, and prophylactic strategies, incidence has been increasing in the last decades, mainly because of the emergence of breakthrough IMI (bIMI) [6]. Although IMI definitions have been clearly established, accurately defining bIMI remains challenging [7–9]. It has been proposed that bIMI should be considered when an IMI is diagnosed after at least 7 days of administration of a mold-active agent, considering that most antifungal drugs will have reached a steady state in that time [8]. Although logical and practical, this definition fails to include the specific characteristics of different antifungal agents, which could have a significant impact on the development of bIMI. Cornely et al. have proposed that bIMI should be defined based on the exposure to a certain antifungal drug, considering the individual pharmacokinetic and other properties of different agents or classes [9]. For instance, time to steady state heavily differs between echinocandins and azoles and also between different azoles, depending on the administration or not of a loading dose, not always administered by clinicians in the setting of antifungal prophylaxis. Considering the frequency of posaconazole as primary or secondary prophylaxis in high-risk hematological patients, as evidenced by existing guidelines and recent surveys, we will predominately discuss posaconazole-associated bIMI in this article, which we believe represent the vast majority of bIMI [10, 11].

## Epidemiology

The incidence of bIMI has been steadily increasing, with recent data suggesting that bIMI represent the majority of IMI amongst allogeneic hematopoeitic cell transplant (HCT) recipients [3, 12]. This can, in part, be attributed to the increasing pool of high-risk hematology patients in the last decades and the routine administration of posaconazole as universal antifungal prophylaxis in patients with acute myelogenous leukemia and allogeneic HCT recipients with acute graft-versus-host disease (GvHD) [10, 13, 14]. Similar to what was observed after the introduction of fluconazole as primary prophylaxis in high-risk patients, with a significant drop in the incidence of invasive candidiasis but the emergence of breakthrough infections due to non-*albicans Candida* spp. and *Aspergillus* spp., the routine use of posaconazole antifungal prophylaxis has led to higher numbers of bIMI. The epidemiology of bIMI is different from primary IMI, often due to pathogens with variable susceptibility profiles and for which treatment options are limited and which were rarely seen in the recent past. For instance, increasing reports have shown the large variety of pathogens associated with bIMI, including non-*fumigatus Aspergillus* spp., such as *A. ustus/callidoustus, A. nidulans, or A. udagawe,* Mucorales (*Rhizomucor pucillus, Rhizopus, Absidia, Cunninghannella*), and other non-Mucorales rare molds: *Fusarium *spp.*, Scedosporium *spp.,* Alternaria *spp.*, Schizophyllum commune, Scopulariopsis spp., Hormographiella aspergillata* [3, 12]. Recent reports suggest that bIMIs may present as either affecting one system, such as sinusitis or lung disease, or as disseminated infection, affecting more than one sites, such as in cases of infections due to the so called “cryptic”-species of *Aspergillus* and other non-*Aspergillus* pathogens [15–17]. Furthermore, bIMIs have been associated with higher mortality rates, when compared to primary IMIs in hematologic patients [12, 17].

## Pathophysiology

Mechanisms leading to bIMI may be related to pharmacokinetic drug characteristics, pathogen, and/or host factors [8, 9].

### Posaconazole Pharmacokinetic Considerations

Suboptimal concentrations of antifungal drugs may play a role in selecting for bIMI. This may be related to suboptimal dosing, suboptimal absorption in the setting of mucositis or gastro-intestinal GvHD, enhanced metabolism due to drug-drug interactions, or still non-adherence to treatment. Therapeutic drug monitoring (TDM) is recommended for patients treated with voriconazole and posaconazole with target trough blood concentrations well defined for both agents [18, 19]. For instance, target trough concentrations for posaconazole have been proposed at > 0.7 and > 1 mg/L for prophylaxis and treatment, respectively [19, 20]. Although those proposed cut-offs are widely accepted and part of consensus guidelines, they are predominately based on post hoc analyses on an exposure–response study on the relationship between posaconazole levels and effective prophylaxis [20]. However, recent data appear to challenge the association between “appropriate” blood trough posaconazole concentrations and effective prophylaxis against IMI. In a single-center study from the US including 343 high-risk patients (adult patients with hematological malignancy receiving posaconazole as primary IMI prophylaxis), eight bIMI were observed [21]. Posaconazole through concentrations were measured in six of them and they were all within the desired target. In another recent multi-center cohort study from the Swiss Transplant Cohort Study (SCTS) including 288 allogeneic HCT recipients who received posaconazole as prophylaxis or treatment, 1944 posaconazole trough levels were measured, 1317 of which in patients who received posaconazole as prophylaxis [22]. Nine (3.1%) breakthrough fungal infections were reported: three with invasive candidiasis and six bIMI. The median time to diagnosis of breakthrough fungal infections was 14 days post posaconazole-initiation. Notably, there was no difference in posaconazole levels between patients with and without breakthrough fungal infections, and in 8/9 patients posaconazole levels were between 0.97 and 1.56 mg/L.

In addition, measuring drug levels in the blood may not necessarily reflect what is actually happening in the target tissue, such as the sinuses or the lungs. First, as posaconazole is a high-protein binding antifungal agent (98–99%), the free plasma posaconazole concentration (or active fraction of the drug) may be subtherapeutic [23]. Second, lung tissue penetration appears to be quite high for posaconazole, reported to concentrate to high levels within cells (up to 40- to 50-fold) and attain therapeutic concentrations in pulmonary epithelial lining fluid and alveolar cells with more than five times the concentration in plasma [24, 25]. Furthermore, posaconazole levels have been found to be high in human peripheral blood mononuclear cells (PBMCs) and polymorphonuclear neutrophils (PMNs) [26]. The above point out the complexity of assessing the efficacy of posaconazole prophylaxis simply depending on blood trough concentrations, an easy and fairly accessible diagnostic tool, which does not necessarily provide clinicians with all pertinent information associated with what actually happens in the tissue on a cellular level and may lead to simplified and -at times-false perceptions.

### Fungal Pathogen Considerations

Breakthrough IMI may be the result of a combination of fungal-associated variables. For instance, the exposure to a high fungal inoculum, which could potentially overcome the protection provided by an effective antifungal prophylaxis and which is not feasible to measure or quantify. In addition, broad-spectrum antifungal prophylaxis may lead to selection for drug-resistant pathogens, such as azole-resistant *Aspergillus* spp. (e.g. *A. ustus/callidoustus*) or other non-*Aspergillus*, non-Mucorales molds (e.g. *Scedosporium* spp., *Lomentospora* spp., *Hormographiella aspergillata*, etc.) [3, 12]. Last, the emergence of azole-resistant *A. fumigatus* reported initially in the Netherlands and Belgium, but currently worldwide, may also lead to even higher rates of bIMI in the future [27].

### Host Factor Considerations

Host factors play an essential role in the pathophysiology of bIMI. As a result of the progress achieved in the field of Hematology and Transplantation, there is a constantly increasing highly-immunocompromised patient population, receiving prolonged courses of immunosuppression, leading to a growing “at risk” population, eventually developing a bIMI. Furthermore, those patients are back in the community, not in isolated positive-pressure hospital rooms anymore, regaining their everyday life activities, and eventually exposed to all types of pathogens, including fungal spore inhalation. In that sense, new forms of treatments for patients with acute myelogenous leukemia, including venetoclax and hypomethylating agents, have as a result large numbers of patients treated as outpatients, with variable neutrophil counts, often neutropenic for prolonged periods of time, without protection against environmental pathogens, such as molds, and without antifungal prophylaxis strategies well defined currently.

### Genetics

Finally, genetic factors may play a role in immune responses to fungal infections. Single nucleotide polymprphisms (SNPs) in toll-like receptors (TLR) 2 or 4 and dectin-1 have been identified as important predisposing factors for infections due to *Aspergillus* spp. in allogeneic HCT recipients [28, 29]. However, the prevalence of specific dectin-1 and TLR2 SNPs in high-risk hematology patients was reported between 3 and 10% [30]. Similarly, the frequency of specific TLR4 haplotypes in donors of allogeneic HCT has been reported in the range of 6% [28]. Initial studies have shown strong associations between some of those polymorphisms and invasive aspergillosis [25, 26, 28, 30]. However, the relatively low prevalence of most of those polymorphisms in the general population may not necessarily allow for meaningful observations and conclusions [29]. In contrast, SNPs in certain pattern recognition receptors (PRR), particularly the pentraxin 3 (PTX3) have been found to have a much higher prevalence in the general population and associated with higher rates of invasive aspergillosis in different patient populations, including allogeneic HCT and solid organ transplant recipients and patients with acute myeloid leukemia [31–34]. Targeting patients harboring those polymorphisms in key-genes, such as that of PTX3, may allow us to stratify antifungal prophylactic strategies based on underlying genetic risk factors and hence decrease the exposure of large patient populations to high-risk antifungal prophylactic agent administration and selection for bIMI. In an ongoing prospective, randomized, stratified, double-blind clinical trial patients with acute myelogenous leukemia receiving intensive chemotherapy are stratified based on the underlying PTX3 SNPs, as high- and low-risk for IMI, and subsequently randomized to either posaconazole or fluconazole (1:1 and 1:3 for the high- and low-risk patients, respectively) [35]. This study enrolls patients in 9 different sites in three European countries and is expected to complete enrollment in 2025.

## Diagnostic and Treatment Considerations

Management of bIMI is challenging, mainly because of the lack of robust evidence to guide clinical decisions and because of the complexity and variety of real-life scenarios. Diagnostics should be performed as routinely recommended for IMI with early imaging (chest computed tomography, CT), serum galactomanan enzyme immunoassay (GM EIA), and a diagnostic procedure, such as bronchoscopy, when feasible. There are some important details in the diagnosis of bIMI, which merit special mention. First, a positive GM EIA may not be adequate enough to guide therapeutic decisions, considering the increasing prevalence of azole-resistant *A. fumigatus* and non-*fumigatus Aspergillus* spp., with variable susceptibility profiles. Second, a negative GM EIA is not adequate enough to rule out a bIMI, considering the high numbers of non-*Aspergillus* bIMI, due to Mucorales and other non-GM producing molds. Third, a bronchoalveolar lavage (or other specimens) on which a fungal culture can be performed is crucial and represents the backbone of the diagnosis, because it may allow for the identification of a fungal pathogen and subsequent susceptibility testing. Fourth, molecular testing using *A. fumigatus*, Mucorales multiplex, or panfungal polymerace chain reaction (PCR) assays on blood and BAL samples may increase the diagnostic yield and allow for the identification of an organism [36, 37]. Finally, antifungal TDM at the time of bIMI diagnosis may be informative and hence still recommended to complete the relevant diagnostic work-up of patients with bIMI.

Antifungal treatment of posaconazole-bIMI includes initiation of empirical treatment with an amphotericin-B formulation, with close clinical, biological, and radiological monitoring of the patient. This empiric approach might allow for treatment of either broad-spectrum resistant *Aspergillus* spp. or Mucorales. In case a pathogen is identified and a susceptibility profile becomes available, antifungal treatment should be tailored accordingly. If a pathogen is identified by PCR and no culture or antibiogram are available, treatment should be adjusted based on susceptibility data from the existing body of literature. Finally, in case that no pathogen is identified, transition from amphotericin-B to another broad-spectrum azole (e.g. isavuconazole) could be considered, after several weeks of empirical treatment and considering the clinical and radiological evolution of the patient. Immunosuppression treatment should be tapered, if and when feasible.

## Conclusion

The incidence of bIMI is increasing in the last years with more rare and challenging fungi identified as significant pathogens, leading to high mortality rates, despite progress in the diagnostic and therapeutic domains. Establishing a microbiological diagnosis remains problematic, with most infections remaining possible or probable, based on PCR rather than culture-based assays. As a result, treatment is based on local epidemiology and expertise, further underscoring the complexity of the management of bIMI. Factors leading to bIMI are variable, predominantly associated with the administration of routine universal broad-spectrum antifungal prophylaxis in high-risk patient populations, changing epidemiology worldwide, and the underlying patient immune status. Recent research efforts, focusing on fully understanding individual susceptibility to IMI and tailoring prophylactic strategies based on underlying genetic risk factors may shed some more light and contribure further to the ongoing battle between host, pathogen, and environment.

## References

[1] Marr KA, Platt A, Tornheim JA, Zhang SX, Datta K, Cardozo C, et al. Aspergillosis complicating severe coronavirus disease. Emerg Infect Dis. 2021;27(1):18–25. 33084566 10.3201/eid2701.202896 PMC7774554

[2] Neofytos D, Horn D, Anaissie E, Steinbach W, Olyaei A, Fishman J, et al. Epidemiology and outcome of invasive fungal infection in adult hematopoietic stem cell transplant recipients: analysis of multicenter prospective antifungal therapy (PATH) alliance registry. Clin Infect Dis. 2009;48(3):265–73. 19115967 10.1086/595846

[3] Roth RS, Masouridi-Levrat S, Chalandon Y, Mamez AC, Giannotti F, Riat A, et al. Invasive mold infections in allogeneic hematopoietic cell transplant recipients in 2020: Have we made enough progress? Open Forum Infect Dis. 2021;9(1):ofab596. 34993259 10.1093/ofid/ofab596 PMC8719608

[4] Roth RS, Masouridi-Levrat S, Giannotti F, Mamez A, Morin S, van Delden C, et al. When and how do we stop antifungal treatment for an invasive mould infection in allogeneic haematopoietic cell transplant recipients? Mycoses. 2022;65(11):1061–7. 35815918 10.1111/myc.13496 PMC9796773

[5] Marr KA, Carter RA, Crippa F, Wald A, Corey L. Epidemiology and outcome of mould infections in hematopoietic stem cell transplant recipients. Clin Infect Dis. 2002;34(7):909–17. 11880955 10.1086/339202

[6] Kontoyiannis DP, Marr KA, Park BJ, Alexander BD, Anaissie EJ, Walsh TJ, et al. Prospective surveillance for invasive fungal infections in hematopoietic stem cell transplant recipients, 2001–2006: overview of the transplant-associated infection surveillance network (TRANSNET) database. Clin Infect Dis. 2010;50(8):1091–100. 20218877 10.1086/651263

[7] Donnelly JP, Chen SC, Kauffman CA, Steinbach WJ, Baddley JW, Verweij PE, et al. Revision and update of the consensus definitions of invasive fungal disease from the European organization for research and treatment of cancer and the mycoses study group education and research consortium. Clin Infect Dis. 2020;71(6):1367–76. 31802125 10.1093/cid/ciz1008 PMC7486838

[8] Lionakis MS, Lewis RE, Kontoyiannis DP. Breakthrough invasive mold infections in the hematology patient: current concepts and future directions. Clin Infect Dis Off Publ Infect Dis Soc Am. 2018;67(10):1621–30. 10.1093/cid/ciy473 PMC6206100 29860307

[9] Cornely O, Hoenig M, Lass-Flör C, Chen SA, Kontoyiannis D, Morrissey C, et al. Defining breakthrough invasive fungal infection–position paper of the mycoses study group education and research consortium (MSG-ERC) and the European Confederation of Medical Mycology (ECMM). Mycoses. 2019;62(9):716–29. 31254420 10.1111/myc.12960 PMC6692208

[10] Tissot F, Agrawal S, Pagano L, Petrikkos G, Groll AH, Skiada A, et al. ECIL-6 guidelines for the treatment of invasive candidiasis, aspergillosis and mucormycosis in leukemia and hematopoietic stem cell transplant patients. Haematologica. 2017;102(3):433–44. 28011902 10.3324/haematol.2016.152900 PMC5394968

[11] Lanternier F, Seidel D, Pagano L, Styczynski J, Mikulska M, Pulcini C, et al. Invasive pulmonary aspergillosis treatment duration in haematology patients in Europe: an EFISG, IDWP-EBMT EORTC-IDG and SEIFEM survey. Mycoses. 2020;63(5):420–9. 32009262 10.1111/myc.13056

[12] Kuster S, Stampf S, Gerber B, Baettig V, Weisser M, Gerull S, et al. Incidence and outcome of invasive fungal diseases after allogeneic hematopoietic stem cell transplantation: a Swiss transplant cohort study. Transpl Infect Dis. 2018;20(6): e12981. 30144374 10.1111/tid.12981

[13] Ullmann AJ, Langston A, Reddy V. Posaconazole or fluconazole for prophylaxis in severe graft-versus-host disease. N Engl J Med. 2007;356(1):335–47. 17251530 10.1056/NEJMoa061098

[14] Cornely OA, Perfect J, Helfgott D, Goh YT, Suresh R. Posaconazole versus fluconazole or itraconazole prophylaxis in patients with neutropenia. N Engl J Med. 2007;356(4):348–59. 17251531 10.1056/NEJMoa061094

[15] Glampedakis E, Erard V, Lamoth F. Clinical relevance and characteristics of *Aspergillus calidoustus* and other aspergillus species of section usti. J Fungi Basel Switz. 2020;6(2):84. 10.3390/jof6020084 PMC7344933 32545485

[16] Lamoth F, Chung SJ, Damonti L, Alexander BD. Changing epidemiology of invasive mold infections in patients receiving azole prophylaxis. Clin Infect Dis. 2017;64(11):1619–21. 28199491 10.1093/cid/cix130

[17] Puerta-Alcalde P, Monzó-Gallo P, Aguilar-Guisado M, Ramos JC, Laporte-Amargós J, Machado M, et al. Breakthrough invasive fungal infection among patients with haematologic malignancies: a national, prospective, and multicentre study. J Infect. 2023;87(1):46–53. 37201859 10.1016/j.jinf.2023.05.005

[18] Patterson TF, Thompson GR, Denning DW, Fishman JA, Hadley S, Herbrecht R, et al. Practice guidelines for the diagnosis and management of aspergillosis: 2016 update by the infectious diseases society of America. Clin Infect Dis. 2016;63(4):e1–60. 27365388 10.1093/cid/ciw326 PMC4967602

[19] Ullmann AJ, Aguado JM, Arikan-Akdagli S, Denning DW, Groll AH, Lagrou K, et al. Diagnosis and management of Aspergillus diseases: executive summary of the 2017 ESCMID-ECMM-ERS guideline. Clin Microbiol Infect. 2018;24:e1–38. 29544767 10.1016/j.cmi.2018.01.002

[20] Jang SH, Colangelo PM, Gobburu JVS. Exposure-response of posaconazole used for prophylaxis against invasive fungal infections: evaluating the need to adjust doses based on drug concentrations in plasma. Clin Pharmacol Ther. 2010;88(1):115–9. 20505665 10.1038/clpt.2010.64

[21] Tverdek FP, Heo ST, Aitken SL, Granwehr B, Kontoyiannis DP. Real-life assessment of the safety and effectiveness of the new tablet and intravenous formulations of posaconazole in the prophylaxis of invasive fungal infections via analysis of 343 courses. Antimicrob Agents Chemother. 2017;61(8):e00188 17. 28507111 10.1128/AAC.00188-17 PMC5527592

[22] Kraljevic M, Khanna N, Medinger M, Passweg J, Masouridi-Levrat S, Chalandon Y, et al. Clinical considerations on posaconazole administration and therapeutic drug monitoring in allogeneic hematopoietic cell transplant recipients. Med Mycol. 2021;59(7):701–11. 33381803 10.1093/mmy/myaa106

[23] Lignell A, Löwdin E, Cars O, Chryssanthou E, Sjölin J. Posaconazole in human serum: a greater pharmacodynamic effect than predicted by the non-protein-bound serum concentration. Antimicrob Agents Chemother. 2011;55(7):3099–104. 21502622 10.1128/AAC.01671-10 PMC3122448

[24] Campoli P, Al Abdallah Q, Robitaille R, Solis NV, Fielhaber JA, Kristof AS, et al. Concentration of antifungal agents within host cell membranes: a new paradigm governing the efficacy of prophylaxis. Antimicrob Agents Chemother. 2011;55(12):5732–9. 21930891 10.1128/AAC.00637-11 PMC3232766

[25] Conte JE, DeVoe C, Little E, Golden JA. Steady-state intrapulmonary pharmacokinetics and pharmacodynamics of posaconazole in lung transplant recipients. Antimicrob Agents Chemother. 2010;54(9):3609–13. 20516276 10.1128/AAC.01396-09 PMC2934990

[26] Farowski F, Cornely OA, Vehreschild JJ, Hartmann P, Bauer T, Steinbach A, et al. Intracellular concentrations of posaconazole in different compartments of peripheral blood. Antimicrob Agents Chemother. 2010;54(7):2928–31. 20457819 10.1128/AAC.01407-09 PMC2897276

[27] Resendiz-Sharpe A, Merckx R, Verweij PE, Maertens J, Lagrou K. Stable prevalence of triazole-resistance in Aspergillus fumigatus complex clinical isolates in a Belgian tertiary care center from 2016 to 2020. J Infect Chemother. 2021;27(12):1774–8. 34518094 10.1016/j.jiac.2021.08.024

[28] Bochud PY, Chien JW, Marr KA, Leisenring WM, Upton A, Janer M, et al. Toll-like receptor 4 polymorphisms and aspergillosis in stem-cell transplantation. N Engl J Med. 2008;359(17):1766–77. 18946062 10.1056/NEJMoa0802629 PMC2656610

[29] Fisher CE, Hohl TM, Fan W, Storer BE, Levine DM, Zhao LP, et al. Validation of single nucleotide polymorphisms in invasive aspergillosis following hematopoietic cell transplantation. Blood. 2017;129(19):2693–701. 28270451 10.1182/blood-2016-10-743294 PMC5428460

[30] Fischer M, Spies-Weisshart B, Schrenk K, Gruhn B, Wittig S, Glaser A, et al. Polymorphisms of dectin-1 and tlr2 predispose to invasive fungal disease in patients with acute myeloid leukemia. PLoS One. 2016;11(3): e0150632. 26963509 10.1371/journal.pone.0150632 PMC4786091

[31] Cunha C, Aversa F, Lacerda João F, Busca A, Kurzai O, Grube M, et al. Genetic PTX3 deficiency and aspergillosis in stem-cell transplantation. N Engl J Med. 2014;370(5):421–32. 24476432 10.1056/NEJMoa1211161

[32] Wójtowicz A, Lecompte TD, Bibert S, Manuel O, Rüeger S, Berger C, et al. PTX3 Polymorphisms and invasive mold infections after solid organ transplant. Clin Infect Dis. 2015;61(4):619–22. 25977268 10.1093/cid/civ386

[33] Brunel AS, Wójtowicz A, Lamoth F, Spertini O, Neofytos D, Calandra T, et al. Pentraxin-3 polymorphisms and invasive mold infections in acute leukemia patients receiving intensive chemotherapy. Haematologica. 2018;103(11):e527–30. 29880612 10.3324/haematol.2018.195453 PMC6278985

[34] Lamoth F, Rubino I, Bochud PY. Immunogenetics of invasive aspergillosis. Med Mycol. 2011;49(Suppl 1):S125–36. 20840014 10.3109/13693786.2010.516408

[35] Pierre-Yves B. PTX3 Genetically Stratified Randomized Double-blinded Allocation Event-driven Clinical Trial for Antifungal Prophylaxis in Patients With Acute Myeloid Leukemia [Internet]. clinicaltrials.gov; 2024 févr [cité 1 janv 2024]. Report No.: NCT03828773. Disponible sur: https://clinicaltrials.gov/study/NCT03828773

[36] Millon L, Caillot D, Berceanu A, Bretagne S, Lanternier F, Morio F, et al. Evaluation of serum mucorales polymerase chain reaction (PCR) for the diagnosis of mucormycoses: the modimucor prospective trial. Clin Infect Dis. 2022;75(5):777–85. 34986227 10.1093/cid/ciab1066

[37] Mah J, Nicholas V, Tayyar R, Moreno A, Murugesan K, Budvytiene I, et al. Superior accuracy of aspergillus plasma cell-free DNA polymerase chain reaction over serum galactomannan for the diagnosis of invasive aspergillosis. Clin Infect Dis. 2023;77(9):1282–90. 37450614 10.1093/cid/ciad420

